# Carbonized Ganoderma Lucidum/V_2_O_3_ Composites as a Superior Cathode for High-Performance Aqueous Zinc-Ion Batteries

**DOI:** 10.3390/molecules29153688

**Published:** 2024-08-04

**Authors:** Guilin Zeng, Zhengda Li, Shaohua Jiang, Wei Zhou

**Affiliations:** 1 Hunan Key Laboratory of Applied Environmental Photocatalysis, Changsha University, Changsha 410022, China; zglhut@163.com; 2 College of Materials and Advanced Manufacturing, Hunan University of Technology, Zhuzhou 412007, China; 3 Jiangsu Co-Innovation Center of Efficient Processing and Utilization of Forest Resources, International Innovation Center for Forest Chemicals and Materials, College of Materials Science and Engineering, Nanjing Forestry University, Nanjing 210037, China; shaohua.jiang@njfu.edu.cn

**Keywords:** V_2_O_3_, carbonized, biomass, cathode, AZIBs, capacity retention

## Abstract

In response to the suboptimal electrochemical performance of low-valence vanadium oxides, Ganoderma lucidum biomass-derived carbon@V_2_O_3_ (V_2_O_3_@CGL) composites were prepared by evaporative self-assembly technology and high-temperature calcination. In the prepared composites, V_2_O_3_ effectively encapsulates CGL, serving as a support for V_2_O_3_ and enhancing electrical conductivity and structural stability. This results in improved overall performance for the composites. They revealed satisfactory electrochemical properties when assembled in aqueous zinc-ion batteries (AZIBs). The preliminary discharge specific capacity of the V_2_O_3_@CGL-2 (VOCG-2) composite electrode reached 407.87 mAh g^−1^ at 0.05 A g^−1^. After 1000 cycles, the capacity retention is 93.69% at 3 A g^−1^. This research underscores the feasibility of employing V_2_O_3_ and abundantly available biomass for high-performance AZIB cathodes.

## 1. Introduction

Severe environmental pollution and energy shortages have compelled us to develop inexpensive and renewable storage devices for energy [1,2]. Lithium-ion batteries, as a type of secondary battery, are currently one of the most widely used energy storage devices on account of their satisfactory energy density and long cycle life [3,4]. Nonetheless, concerns about safety and limited resources, such as lithium metal, have driven the exploration of new battery systems. In recent years, water-based metal-ion batteries (e.g., zinc, sodium, potassium, magnesium, and calcium) have shown enormous possibilities in energy storage, considering the abundant reserves of metal resources on earth and their inherent safety [5].

Among these options, AZIBs have garnered significant curiosity from researchers worldwide because of their rich sources, non-toxicity, high safety, low REDOX potential (−0.76 V), and excellent theoretical capacity (approximately 820 mAh g^−1^) [6,7,8,9]. However, research on AZIBs is still in its early stages, and it is challenging to find positive electrode materials suitable for reversible Zn^2+^ embedding or de-embedding, restricting the development of AZIB systems. Previous studies on AZIB cathode materials have focused on Prussian blue analogues with a cubic open frame structure [10]. However, this has a limited capacity (about 60 mAh g^−1^), which hinders further development. Manganese oxides, such as MnO_2_, α-Mn_2_O_3_, and Mn_3_O_4_, have considerable voltage and desirable capacity. Nonetheless, the dissolution of manganese in the electrolyte leads to poor cycling performance [11,12,13].

Among AZIB cathode materials, vanadium-based materials have been extensively researched for their high specific capacity, vast resources, and excellent cycle stability [14,15,16,17]. For instance, Hu et al. [18] obtained porous V_2_O_5_ material (P-V_2_O_5_) by pyrolyzing V-MOF. As the cathode for AZIBs, the P-V_2_O_5_ electrode manifested a capacity of 120 mAh g^−1^ at 2 A g^−1^. They also demonstrated that the formation of V_2_O_5_ nanoflakes and the reorganization with carbon can increase cycle stability. Mai et al. [19] successfully developed Na_2_V_6_O_16_·1.63H_2_O material that is highly suitable for Zn^2+^ embedding and removal, demonstrating a noteworthy specific capacity of 352 mAh g^−1^ and a desirable long-cycle stability with a capacity retention of 90% for 6000 cycles at 0.05 A g^−1^.

Although well developed in small-scale production, the above synthesis methods are still far from commercialization due to their complexity, the high cost of electrolyte and electrode materials, and unsustainable carbon sources [20,21]. Therefore, the search for cheap, abundant, and renewable raw materials gradually gains popularity. Biomass, a carbon-rich precursor, has been extensively researched in various applications because of its inherent benefits, such as environmental friendliness, abundant renewable resources, and economic benefits [22,23,24].

In this study, the Ganoderma lucidum biomass was first activated using KOH ultrasonic solvent and calcination. The observed V_2_O_3_@CGL composites exhibited a large specific surface area and numerous mesopores, which furnished abundant active sites and efficient channels for reversible storage of Zn^2+^. Three Ganoderma lucidum biomass-derived carbon/V_2_O_3_ composites were prepared as positive electrodes for AZIBs, with the economical 3M ZnSO_4_ serving as the electrolyte. The VOCG-2 composite electrode displayed outstanding durability with a satisfactory capacity retention of 93.69% after 1000 cycles at 3 A g^−1^. SEM analysis confirmed that the VOCG-2 composite electrode maintained a steady morphology during circulation. These findings suggest VOCG-2 composites offer promising potential for fast and long-lasting storage of Zn^2+^.

## 2. Results

**Figure 1:**
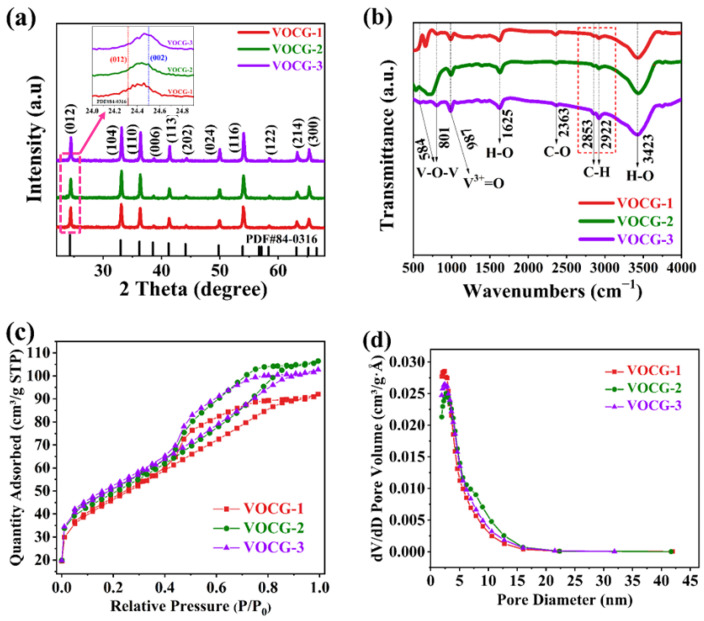
(**a**) XRD patterns; (**b**) FT-IR spectrum; (**c**) N_2_ absorption/desorption isotherms; and (**d**) pore size distribution of the V_2_O_3_@CGL composites. Inset images show the (012) and (002) diffraction planes of the V_2_O_3_@CGL composites.

Through XRD investigations, detailed information regarding the crystal structure of the V_2_O_3_@CGL composites was obtained. Figure 1a illustrates the XRD patterns of the V_2_O_3_@CGL composites, displaying a broad diffraction peak at approximately 2θ = 24.5°, which is consistent with the (002) plane of amorphous carbon [25]. The peak intensity of this diffraction peak diminishes with the reduction of carbon in the composites. No additional noteworthy residual phases were detected, implying that there is no noticeable impact of CGL on the crystal structure of V_2_O_3_. In addition, the diffraction peaks centered at 65.2°, 53.9°, 41.3°, 36.3°, 33.0°, and 24.3°, correspond to the (300), (116), (113), (110), (104), and (012) diffraction planes, respectively, of the rhombic crystalline phase of V_2_O_3_ (PDF#84-0316), evidencing the successful synthesis of the V_2_O_3_ phase.

Figure 1b reveals a typical FT-IR spectrum of the V_2_O_3_@CGL composites. The peaks situated at 2853 and 2922 cm^−1^ denote the presence of residual C-H groups [26]. The peak observed at 2367 cm^−1^ corresponds to an asymmetric stretching vibration of C-O, which is attributed to CO_2_ adsorption on KBr and is negligible [27]. The H-O bending vibration and H-O stretching vibration can be identified at 1625 and 3423 cm^−1^, respectively, which may be because of certain water molecules adsorbed on the surface and embedded in the composite interlayers [28]. Furthermore, the peaks located at 801 and 584 cm^−1^ are ascribed to the symmetric and asymmetric stretching vibrations of the V-O-V bond [29,30,31]. The signal at 987 cm^−1^ is related to the symmetric stretching of V^3+^=O, suggesting the presence of V_2_O_3_ [32,33]. Based on the above analysis, the synthesized V_2_O_3_@CGL composites consist of V_2_O_3_ and biomass-derived carbon, which coincides with the results of the XRD.

**Figure 2:**
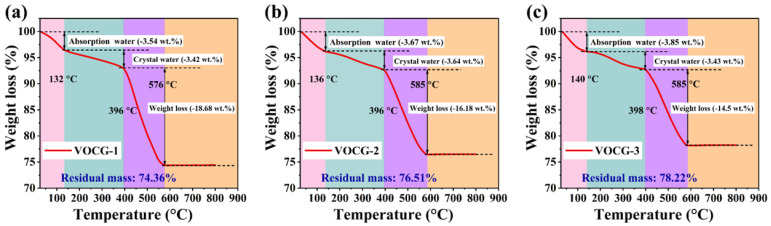
TGA curves of (**a**) VOCG-1, (**b**) VOCG-2, and (**c**) VOCG-3 composites.

The porosity and specific surface area of electrode materials are critical for ion diffusion. Therefore, N_2_ adsorption/desorption isotherm experiments were carried out. As depicted in Figure 1c,d, the three V_2_O_3_@CGL composites exhibit typical IV isotherms followed by H_3_-type hysteresis loops, suggesting that the materials include a significant number of mesoporous pores in the samples. The average pore diameter, pore volume, and specific surface area of the three V_2_O_3_@CGL composites are summarized in Table 1. Among them, the specific surface area of VOCG-3 composite is as high as 174.2683 cm^2^ g^−1^, which is significantly larger than VOCG-1 (154.9935 cm^2^ g^−1^) and VOCG-2 (164.5602 cm^2^ g^−1^), suggesting that with a higher content of V_2_O_3_, the specific surface area will increase. Additionally, the pore sizes of the three V_2_O_3_@CGL composites range from 2 to 43 nm (see Figure 2d). The abundant mesoporous structure and large specific surface area facilitate rapid storage of Zn^2+^. Furthermore, a suitable pore size distribution promotes ion diffusion, thereby enhancing the magnification performance of V_2_O_3_@CGL composites [34].

To ascertain the weight percentage of every ingredient in the V_2_O_3_@CGL composites, a TGA test was carried out at 25–800 °C in nitrogen, as illustrated in Figure 2. The three TGA curves exhibit three distinct weightlessness stages. The first two stages occur at room temperature to about 136 °C and 400 °C, respectively, which are associated with the release of structural water and adsorbed water in the V_2_O_3_@CGL composites. The mass loss in the first two stages of VOCG-1, VOCG-2, and VOCG-3 was 6.96%, 7.31%, and 7.28%, respectively. The third stage of weightlessness occurs at about 400–580 °C, which is related to the combustion of CGL in the V_2_O_3_@CGL composites. The weightlessness in the third stage for VOCG-1, VOCG-2, and VOCG-3 composites was 18.68%, 16.18%, and 14.5%, respectively. According to the data obtained from the TGA, the mass content of CGL and V_2_O_3_ in VOCG-1, VOCG-2, and VOCG-3 was 18.68% and 74.36%, 16.18% and 76.51%, and 14.5% and 78.22%, respectively.

**Figure 3:**
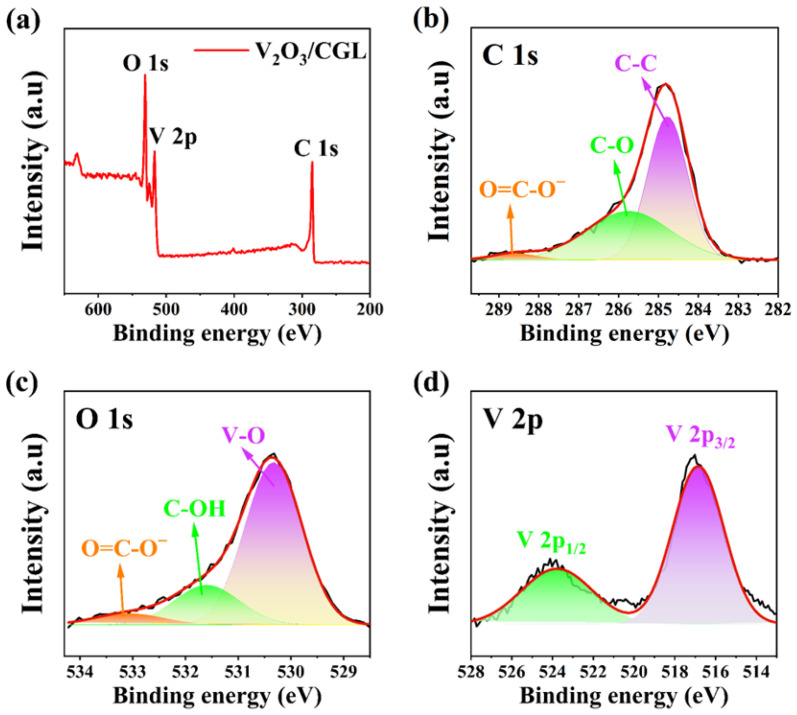
(**a**) XPS survey spectra; at high-resolution: (**b**) C 1s, (**c**) O 1s, and (**d**) V 2p XPS spectra of the V_2_O_3_@CGL composites.

The surface elemental composition, electronic states, and bonding states of the V_2_O_3_@CGL composites were studied by means of XPS spectroscopy. As depicted in Figure 3a, V, O, and C elements were detected in the XPS measurement spectra. The bonding state of V, C, and O was further evaluated by the V 2p, C 1s, and O 1s peaks. As displayed in Figure 3b, the peaks are located at 288.58, 285.76, and 284.77 eV, respectively, matching the O=C-O^−^, C-O, and C-C bonds of C 1s, which are derived from CGL [35,36,37]. Figure 3c exhibits three contributions from the fitted O 1s peaks, with binding energies of 533.06, 531.6, and 530.33 eV, attributed to the O=C-O^−^, C-OH, and V-O bonds [22,38,39], respectively. The V 2p peak of the V_2_O_3_@CGL composites (Figure 3d) is decomposed into two peaks at 523.78 and 516.8 eV, corresponding to V 2p_1/2_ and V 2p_3/2_, proving the presence of V_2_O_3_ [40,41,42].

**Figure 4:**
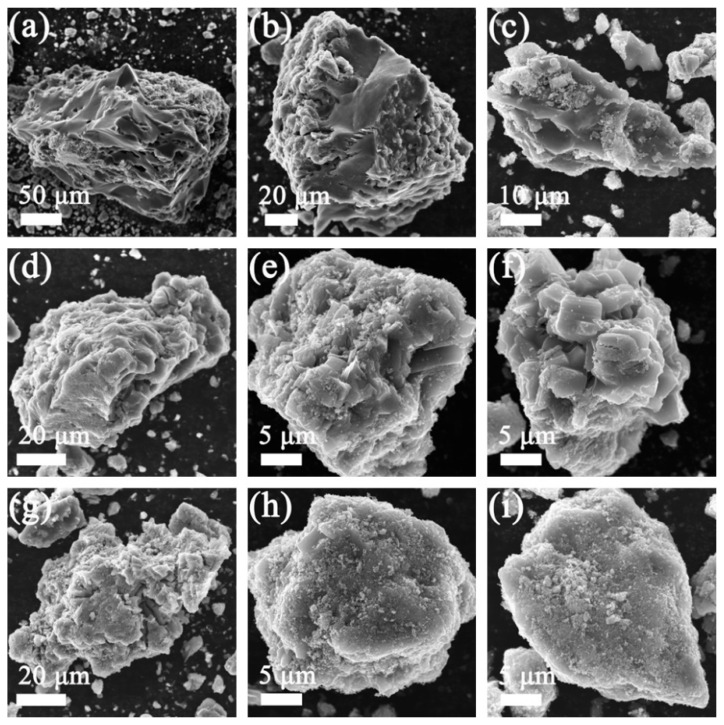
SEM pictures of (**a**–**c**) VOCG-1, (**d**–**f**) VOCG-2, and (**g**–**i**) VOCG-3 composites.

**Figure 5:**
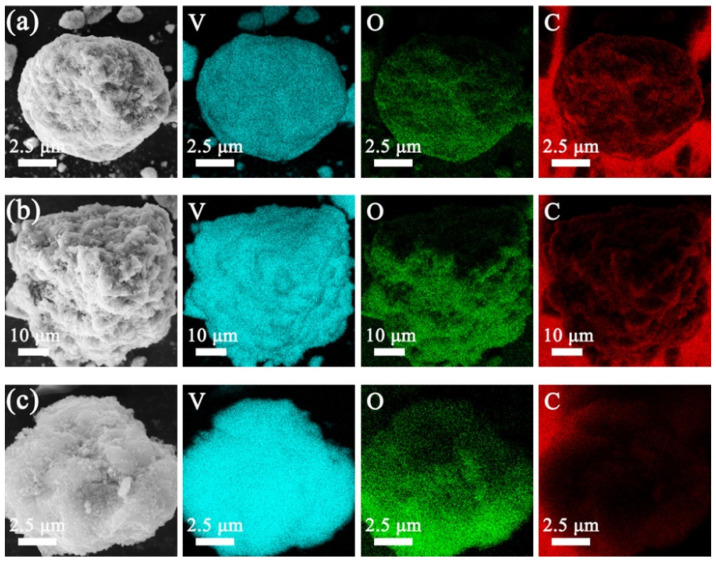
SEM and elemental mapping images of (**a**) VOCG-1, (**b**) VOCG-2, and (**c**) VOCG-3 composites.

Figure 4 illustrates the SEM pictures of the V_2_O_3_@CGL composites. The CGL in the V_2_O_3_@CGL composites reveals an irregular three-dimensional porous structure with diameters ranging from 30 to 300 μm. V_2_O_3_ is observed to be encapsulated on the surface of the CGL or entering its pores. The surface of VOCG-1 composite is relatively smooth, with the exposed Ganoderma lucidum biomass-derived carbon visible, while the surfaces of VOCG-2 and VOCG-3 composites are relatively rough. Notably, VOCG-3 is completely covered by V_2_O_3_, with the bare Ganoderma lucidum biomass-derived carbon barely discernible. This indicates that VOCG-1 composite contains the least amount of V_2_O_3_, while VOCG-3 composite contains the greatest amount of V_2_O_3_. Furthermore, CGL can provide a carbon skeleton for V_2_O_3_, effectively overcoming the adverse effects of V_2_O_3_ aggregation and volume expansion during charging and discharging, thus enhancing the zinc storage performance [43]. Moreover, the elemental mapping of the V_2_O_3_@CGL composites is presented in Figure 5, which reveals that the C, O, and V elements are homogeneously dispersed in the V_2_O_3_@CGL composites. This, together with the XRD and XPS data presented above, provides evidence that the synthesis of the V_2_O_3_@CGL composites was successful. It is notable that a comparison of the brightness of the elemental maps of the three composites reveals that the VOCG-1 composite has the highest concentration of carbon and the lowest concentration of vanadium, while the VOCG-3 composite has the lowest concentration of carbon and the highest concentration of vanadium. This indicates that the VOCG-1 composite has the lowest vanadium pentoxide content, while the VOCG-3 composite has the highest V_2_O_3_ content.

**Figure 6:**
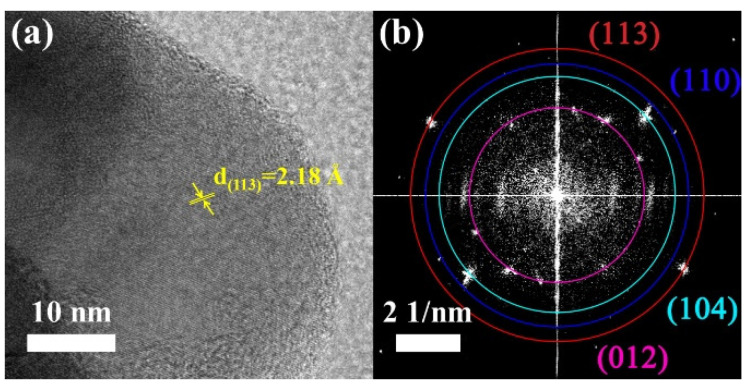
(**a**) HRTEM map and (**b**) SAED diagram of the V_2_O_3_@CGL composites.

The HRTEM map and the corresponding selected area electron diffraction (SAED) diagram of the V_2_O_3_@CGL composites are presented in Figure 6. The majority of the lattice fringes of the V_2_O_3_@CGL composites are more pronounced. The corresponding crystallographic spacing is approximately 2.18 Å, which is consistent with the (113) crystallographic plane of V_2_O_3_, thereby confirming the presence of V_2_O_3_ in the V_2_O_3_@CGL composites. Furthermore, the SAED diagram of the V_2_O_3_@CGL composites is presented in Figure 6b. The presence of significant diffraction rings at the (012), (104), (110), and (113) facets of V_2_O_3_ was observed, which was in accordance with the XRD results. This once again demonstrates that V_2_O_3_ exhibits excellent crystallinity.

## 3. Discussion

**Figure 7:**
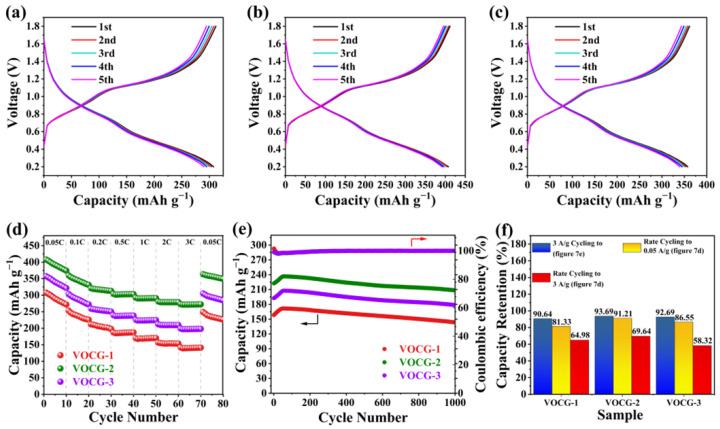
GCD profiles of (**a**) VOCG-1, (**b**) VOCG-2, and (**c**) VOCG-3 composites in the original five cycles; (**d**) rate; (**e**) cycling properties; and (**f**) capacity retention after 1000 cycles at 3 A g^−1^ (blue), capacity retention after rate cycling to 3 A g^−1^ (yellow) and rate cycling back to 0.05 A g^−1^ (red) of the three samples.

GCD measurements were performed on the V_2_O_3_@CGL composite electrodes within the range 0.2–1.8 V at 0.05 A g^−1^. The resulting GCD profiles for the first five turns are presented in Figure 7a–c. The two pairs of redox voltage plateaus observed at 0.58/0.88 and 0.98/1.21 V on both the charge and discharge curves correspond to the CV curves below. The initial discharge specific capacity of the VOCG-2 composite electrode can be observed to reach 407.88 mAh g^−1^, which is considerably greater than that of VOCG-1 (307.64 mAh g^−1^) and VOCG-3 (357.43 mAh g^−1^). Although only V_2_O_3_ provides the specific capacity in the composites, a high proportion of V_2_O_3_ does not necessarily exhibit the highest specific capacity. This is due to the poor structural stability and intrinsic lack of electrical conductivity of V_2_O_3_. Therefore, the discharge specific capacity of V_2_O_3_ can be effectively optimized by the addition of an appropriate amount of CGL.

Figure 7d illustrates the rate capability of the V_2_O_3_@CGL composite electrodes at varying current densities. The current density is incrementally raised from 0.05 C to 3 C and subsequently decreased to 0.05 C (specific multiplicity values are listed in Figure 7d). The discharge specific capacity exhibited a gradual decline as the current density increased. The average discharge specific capacities of VOCG-2 were 344.48, 316.61, 302.85, 290.84, 279.03, and 272.00 mAh g^−1^, respectively, which were significantly higher than those of VOCG-1 and VOCG-2. This indicates that VOCG-2 is superior in multiplicity performance. Upon the return of the current density to 0.05 C, the discharge specific capacity of VOCG-2 also recovered to 356.23 mAh g^−1^, which was 91.21% of the initial value. In contrast, the discharge specific capacity of VOCG-1 was only 81.33% (235.17 mAh g^−1^) and that of VOCG-3 was 86.55% (293.50 mAh g^−1^) of the initial value. The VOCG-1 and VOCG-2 electrodes exhibited a capacity of only 81.33% (235.17 mAh g^−1^) and 86.55% (293.50 mAh g^−1^) of the initial value, respectively. The aforementioned outcomes demonstrate that the VOCG-2 electrode exhibits excellent reversibility. Moreover, the crystal structure of the VOCG-2 electrode exhibits enhanced stability. The exceptional multiplicity performance of VOCG-2 may be attributed to the incorporation of CGL, which enhances the structural stability and conductivity of the electrode, facilitating the rapid (de)intercalation of carriers.

Figure 7e illustrates the cycling properties of the three V_2_O_3_@CGL composites at 3 A g^−1^. The capacities of all three composite electrodes exhibited a gradual increase over the initial 60 cycles, in agreement with vanadium-based materials reported in the literature, and may be related to the gradual electrochemical activation. The first discharge specific capacity of the VOCG-2 composite electrode was 222.41 mAh g^−1^, which was considerably superior to that of the VOCG-1 (158.32 mAh g^−1^) and VOCG-3 (192.55 mAh g^−1^) composite electrodes. After 56 cycles, the specific capacity of the VOCG-2 electrode reached a maximum of 236.71 mAh g^−1^. However, the maximum discharge specific capacities of the VOCG-1 and VOCG-3 composite electrodes were only 171.69 and 207.68 mAh g^−1^, respectively, after 53 and 58 cycles, which were significantly lower than that of the VOCG-2 electrode. Moreover, the reversible specific capacity of the VOCG-2 electrode was obtained at 208.38 mAh g^−1^ after 1000 cycles, with a capacity retention of 93.69%. In contrast, the specific capacities of the VOCG-1 and VOCG-3 electrodes were somewhat lower, at 143.51 mAh g^−1^ and 178.46 mAh g^−1^, respectively. Moreover, the capacity retentions were not as good as those of VOCG-2, at 90.65% and 92.67%, respectively. Consequently, the VOCG-2 electrode exhibits superior cycling stability. The exceptional electrochemical property of the VOCG-2 electrode is attributed to the CGL, which not only improves the electrode’s conductivity but also provides a well-developed pore structure that facilitates the diffusion of ions, thereby ensuring an optimal ion diffusion rate.

**Figure 8:**
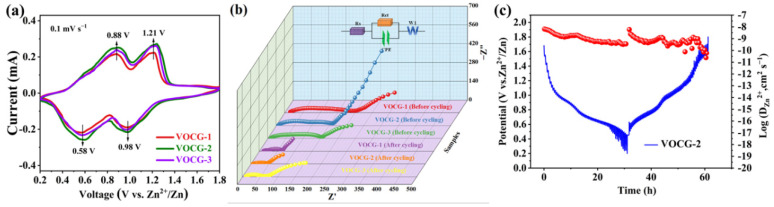
(**a**) CV curves of VOCG-1, VOCG-2, and VOCG-3 composites before and after cycling; (**b**) EIS spectra of the V_2_O_3_@CGL composite cathodes before and after cycling; and (**c**) GITT curve and corresponding D_Zn_^2+^ values for the VOCG-2 composite cathode.

The CV curves were utilized to evaluate the electrochemical process kinetics of the V_2_O_3_@CGL electrode within the range 0.2–1.8 V, as depicted in Figure 8a. The three CV curves possess similar shapes, with two pairs of distinct coupled REDOX peaks placed at about 0.58/0.88 V and 0.98/1.21 V, respectively, indicating that the insertion of Zn^2+^ in the V_2_O_3_@CGL electrode undergoes a two-step reversible reaction, akin to the previously reported vanadium-based cathodes [6,16,34]. It has been reported that the area and current depicted in the CV curve are closely linked to the obtained capacity [44]. It is easily observed that the area of the VOCG-2 electrode is the largest, while the VOCG-1 electrode has the smallest area. Consequently, the VOCG-2 electrode has the largest specific capacity among them, while the VOCG-1 capacity is relatively lower.

To further evaluate the charge transfer state of the V_2_O_3_@CGL composite electrodes, EIS measurements were carried out, and the corresponding Nyquist and EIS plots are depicted in Figure 8b. The three EIS curves exhibit a semicircle at medium-high frequencies and a straight line at low frequencies. The semicircle reflects charge transfer resistance (R_ct_), while the straight line is related to the ion diffusion process (R_s_) within the electrode [45,46]. The equivalent circuit presented in Figure 8b was utilized for fitting, and the detailed fitting data are summarized in Table 2. Notably, the R_ct_ values of the VOCG-2 composite electrode pre- and post-cycling were significantly lower than those of VOCG-1 and VOCG-3, indicating the superior electrical conductivity of the VOCG-2 electrode. Furthermore, the R_ct_ values for all three V_2_O_3_@CGL electrodes after cycling are notably smaller compared to those before cycling, suggesting improved charge transfer kinetics following multiple cycles.

To accurately investigate the diffusion kinetics of Zn^2+^ (D_Zn_^2+^) in the VOCG-2 composite electrode, GITT measurements were performed, and D_Zn_^2+^ was calculated using Formula (1):

(1) $$D=\frac{4L^{2}}{\pi \tau }(\frac{{\Delta E}_{s}}{{\Delta E}_{t}}{)}^{2}$$

where ΔE_t_ is the change in voltage during the continuous current pulse after the i_R_ drop has been removed and ΔE_s_ is the change in steady-state potential owing to the current pulse. The electrode thickness is denoted as L, while the relaxation time is represented by τ. The GITT profile and the calculated D_Zn_^2+^ values of the VOCG-2 composite electrode are depicted in Figure 8c. It can be observed that the D_Zn_^2+^ values of the VOCG-2 composite electrode are in the range of 10^−10.5^ and 10^−8^ cm^2^ s^−1^ during cycling, which is a relatively good level. This indicates that Zn^2+^ has satisfactory diffusion kinetics in the VOCG-2 electrode, which is mainly associated with the natural porous structure of CGL, which can shorten the diffusion path for Zn^2+^ transport and promote its effective transport.

**Figure 9:**
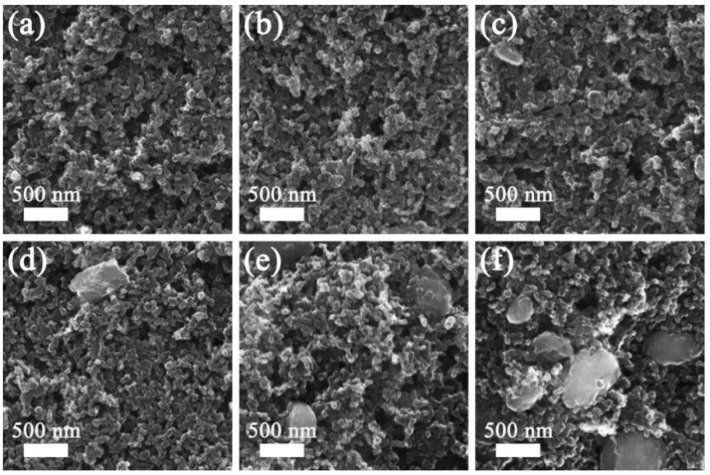
SEM photographs of the VOCG-2 composite electrodes at various stages: (**a**) pristine, (**b**) 200, (**c**) 400, (**d**) 600, (**e**) 800, and (**f**) 1000 cycles.

The morphological evolution of the VOCG-2 composite electrode in the pristine state and at different stages was investigated by SEM, as revealed in Figure 9a–f, respectively. The nanoparticles were evenly arranged on the stainless steel foil without agglomerating in their pristine state. After 200 and 400 cycles, the VOCG-2 composite electrode presented little morphological change, suggesting excellent structural stability during cycling. After 600 cycles, slight pulverization and agglomeration appeared on the surface of the VOCG-2 electrode. As charging and discharging continued, the pulverization and agglomeration were more pronounced (see Figure 9e,f), corresponding to the decrease in capacity in Figure 8e. Notably, no cracks or obvious dendrites appeared from the initial state to 1000 cycles (see Figure 9a–f), disclosing the protective mechanism of the array structure of the CGL. Therefore, the resulting VOCG-2 composite has good structural stability, which is advantageous for enhancing the cycle lifetime of AZIBs.

Table 3 summarizes the electrochemical properties of several previously reported vanadium-based cathodes utilized for AZIB applications. The results reveal that the VOCG-2 composite proposed in this paper has certain advantages, and the desirable electrochemical properties of the VOCG-2 composite electrode can be correlated with the appropriate CGL content to increase the structural stability and electrical conductivity. Furthermore, the plentifully mesoporous structure and large specific surface area of CGL can facilitate the rapid storage of Zn^2+^.

## 4. Experimental Section

### 4.1. Preparation of V_2_O_3_@CGL Composites

The Ganoderma lucidum was repeatedly cleaned with distilled water to remove soil, and placed in a drying oven at 60 °C until it was completely dry. The dried Ganoderma lucidum and KOH were mixed according to the mass ratio of 1:4 with deionized water as the ultrasonic solvent for two hours. It was then transferred to a blast drying oven maintained at 80 °C for the purpose of complete drying, followed by calcination at 600 °C for 2 h in argon to acquire Ganoderma lucidum biomass-derived carbon (CGL).

**Figure 10:**
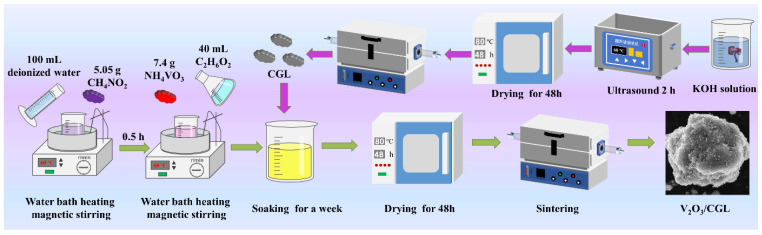
The preparation process of the V_2_O_3_@CGL composites.

The detailed synthesis procedure for the V_2_O_3_@CGL composites is displayed in Figure 10. Firstly, 5.05 g CH_4_NO_2_ and 7.4 g NH_4_VO_3_ were dissolved in 100 mL of distilled water, followed by stirring in a water bath at 60 °C for 0.5 h. Subsequently, 40 mL of C_2_H_6_O_2_ solution and 0.3 g of CGL were mixed into the above solution, sealed, and left for a week. In order to completely evaporate the water, the mixture was dried in an oven at 80 °C for 48 h. The final stage of the process involved the transfer of the resulting precursors to a corundum crucible and their placement in a tubular furnace. Subsequently, the furnace was heated to 350 °C for 4 h and then heated to 800 °C for 8 h at a rate of 5 °C min^−1^ in an argon environment. This procedure yielded the desired VOCG-3 composite. The mass ratio of NH_4_VO_3_ to chestnut needle was adjusted in order to prepare the VOCG-1 and VOCG-2 composites by the same method. Table 4 illustrates the quality of the raw materials produced for each sample.

### 4.2. Material Characterization

Detailed information on XRD, TGA, XPS, SEM, HRTEM, and FT-IR testing can be found in our previous paper: 10.3390/molecules28052147 [47]. The isothermal nitrogen adsorption/desorption test was conducted on the samples using an ASAP 2020 tester from Micromeritics, Norcross, GA, USA, maintained at 77 K beneath liquid nitrogen.

### 4.3. Electrochemical Measurements

In order to prepare the cathode, PVDF (10 w.t.%), acetylene black (20 w.t.%) and active material (70 w.t.%) were successively dispersed in N-methyl-2-pyrrolidone. The resulting mixed slurry was evenly coated on stainless steel foil and dried in a vacuum at 60 °C for 12 h. A CR2025 coin battery was assembled in air with glass fiber adopted as the diaphragm, 3 M ZnSO_4_ aqueous solution employed as the electrolyte, and commercial zinc foil utilized as the anode. For detailed information on partial electrochemical testing, please refer to our previous paper [47]. The galvanostatic intermittence titration technique (GITT) was carried out using the NETWARE test instrument within the range 0.2–1.8 V.

## 5. Conclusions

In this study, the V_2_O_3_@CGL composites were prepared using evaporation self-assembly technology with Ganoderma lucidum as the carbon source and NH_4_VO_3_ as the metal source. In these V_2_O_3_@CGL composites, CGL exhibits a porous structure and V_2_O_3_ provides large capacity, which can increase the electrolytic/cathodic contact area and provide incremental active sites. Moreover, the introduction of CGL increases the mechanical properties, while also making up for V_2_O_3_’s inadequate electrical conductivity. Thus, the V_2_O_3_@CGL composites possess the ideal electrochemical properties. Specifically, the VOCG-2 composite demonstrated superior initial discharge specific capacity and excellent cycle stability. Furthermore, SEM testing revealed that the VOCG-2 electrode microstructure remained stable without obvious cracks or zinc dendrites during cycling, which contributes to its excellent zinc storage properties. This research introduces an innovative strategy for the enhancement of the electrochemical properties of V_2_O_3_ and these results will assist in creating affordable high-performance vanadium-based AZlBs.

## Figures and Tables

**Table 1:** Pore volume, specific surface area, and average pore size of the V_2_O_3_@CGL composites.

Sample	Pore Volume (cm^3^ g^−1^)	Specific Surface Area (m^2^ g^−1^)	Average Pore Size (Å)
VOCG-1	0.1424	154.9935	3.6760
VOCG-2	0.1647	164.5602	4.0040
VOCG-3	0.1589	174.2683	3.6465

**Table 2:** Electrochemical impedance spectra of the three V_2_O_3_@CGL composites before and after cycling.

Sample	VOCG-1	VOCG-2	VOCG-3
R_ct_ (before cycling)	180.8 Ω	129.8 Ω	141 Ω
R_ct_ (after cycling)	63.47 Ω	40.92 Ω	55.29 Ω
R_s_ (before cycling)	4.04 Ω	2.56 Ω	3.73 Ω
R_s_ (after cycling)	7.72 Ω	3.72 Ω	4.48 Ω

**Table 3:** Comparison of the electrochemical properties of the V_2_O_3_@CGL composite with other vanadium-based AZIB cathode materials that have been previously reported in the literature.

Sample	Cycle Number	Capacity Retention	Current Density (A g^−1^)	Specific Capacity (mAh g^−1^)	Ref.
VOCG-2	1000	93.69%	3	208.38	This work
V_2_O_3_@carbonized Dictyophora	1000	89.24%	1	151.9	[47]
V_2_O_3_/carbonized chestnut needle	1000	94.26%	3	213.66	[48]
V_2_O_3_	100	76.9%	0.1	161	[34]
Polyaniline-intercalated V_2_O_5_@nH_2_O	100	57%	0.1	196	[7]
Mn_0.31_V_3_O_7_@1.40H_2_O	500	54%	1	164	[49]
(NH_4_)_x_V_2_O_5_@nH_2_O	50	63%	0.1	235	[50]
V_2_O_x_@V_2_CT_x_	200	81.6%	1	87.3	[51]
V_2_O_3_@carbon nanofibers	1000	80%	0.2	120	[39]
V_6_O_13_@hollow carbon microspheres	1000	76%	1	162.1	[52]
Carbon-coated NaVPO_4_F	400	94.5%	0.1	87.4	[53]
V_2_O_3_@amorphous carbon	1600	90.7%	1	116	[6]
V_2_O_3_@rGO	1000	114%	5	195	[54]
VO_2_ hollow nanospheres	860	47.6%	1	143	[15]
δ-Na_x_V_2_O_5_/VO_2_(B)	200	94%	4	253	[55]
FeVO_4_·nH_2_O@rGO	1000	43.8%	1	92	[12]

Values are estimated from the graphs.

**Table 4:** Summary of the dosage of raw materials synthesized from each sample.

Sample	CGL	NH_4_VO_3_	CH_4_NO_2_	C_2_H_6_O_2_	H_2_O
VOCG-1	0.3 g	5.04 g	3.43 g	40 mL	100 mL
VOCG-2	0.3 g	6.24 g	4.26 g	40 mL	100 mL
VOCG-3	0.3 g	7.40 g	5.05 g	40 mL	100 mL

## Data Availability

Data will be made available on request.
